# Comprehensive Analysis on the Specific Role and Function of Mitochondrial Inner Membrane Protein MPV17 in Liver Hepatocellular Carcinoma

**DOI:** 10.1155/2022/7236823

**Published:** 2022-07-19

**Authors:** Hanchuan Tao, Cheng Wang, Chongmei Lu, Ning Ma, Yifan Zhu, Shihai Xuan, Xiaojun Zhou

**Affiliations:** ^1^Department of General Surgery, First Affiliated Hospital of Soochow University, Soochow, Jiangsu 215006, China; ^2^Department of General Surgery, Dongtai People's Hospital, Yancheng, Jiangsu 224200, China; ^3^Department of Neurology, Dongtai People's Hospital, Yancheng, Jiangsu 224200, China; ^4^Department of General Surgery, Daqing Oilfield General Hospital, Daqing 163001, Heilongjiang, China; ^5^Department of Basic Medicine Kangda College of Nanjing Medical University, Nanjing 210028, Jiangsu, China; ^6^Department of Clinical Laboratory, Dongtai People's Hospital, Yancheng, Jiangsu 224200, China

## Abstract

**Background:**

Liver hepatocellular carcinoma (LIHC) is the predominant type of liver cancer, and its treatment still faces great challenges presently. Mitochondrial inner membrane protein MPV17 is reported to be involved in multiple biological activities of cancers. Here, we seek to investigate the specific role and functions of MPV17 in LIHC progression.

**Methods:**

Firstly, MPV17 expressions in various tumors and corresponding normal samples and LIHC groups with various clinical features were analyzed, respectively. Next, the relationship between MPV17 expression and LIHC survival was analyzed and verified by AUC curves. Besides, differentially expressed genes (DEGs) for LIHC were screened from TCGA and then analyzed by GO and KEGG. Then, MPV17 was analyzed by prognostic model, Cox analysis, predictive nomogram, pathway correlation, and immunoassay. Finally, the functions of MPV17 were determined by CCK-8 and Tranwell assays.

**Results:**

In most tumors, MPV17 expression was higher than that in the normal group, and it was related to LIHC clinical features. In the LIHC survival analysis, highly expressed MPV17 was associated with a poor prognosis. Besides, 314 upregulated and 193 downregulated DEGs are mainly involved in the TNF signaling pathway and tyrosine metabolism. Through prognostic model, Cox analysis, and predictive nomogram, MPV17 had the prognostic value for LIHC. Gene-pathway correlation analysis showed that MPV17 had the strongest correlation with the G2M_checkpoint pathway. In an immunoassay, MPV17 had a strong correlation with many immune cells. Functional assays showed that MPV17 reduction in LIHC cells could inhibit cell invasion, migration, and proliferation.

**Conclusion:**

MPV17, as a tumor promoter, could be a new biomarker for LIHC diagnosis and prognosis and probably shed new light on the exploration of LIHC therapies.

## 1. Background

Globally, liver carcinoma is the fourth most widely incurred inducer of carcinoma-correlated mortality, ranking sixth amongst emergency events [1]. Liver hepatocellular carcinoma (LIHC) is the main subtype of primary liver carcinoma, accounting for up to approximately 90% of cases [2, 3]. Presently, LIHC treatment faces huge difficulties [4, 5]. On the one hand, the early symptoms of LIHC are atypical, and most LIHC patients get diagnosed at an advanced stage [6]. On the other hand, due to the high LIHC metastasis and recurrence, the prognosis of patients after liver transplantation treatment is still poor [7, 8]. Understanding the molecular mechanism of LIHC will facilitate biological insights and the detection of novel therapeutic targets. However, the occurrence and development of LIHC have not been clear exactly [9].

As an inner membrane protein of mitochondrial, mitochondrial inner membrane protein MPV17 (MPV17) has a ubiquitous expression in adrenal and thyroid. Presently, it still remains elusive in many aspects, such as cell activities and cancer development. So far, it is reported to be involved in the reactive oxygen species (ROS) metabolic process [10–12]. Zwacka et al. revealed that MPV17 protein had a function in peroxisomal reactive oxygen metabolism, and a new relationship between peroxisomal ROS generation and glomerulosclerosis was discovered [13]. Besides, some studies have shown that the mutated MPV17 gene leads to a mitochondrial DNA depletion syndrome of human hepatocerebral [10, 14, 15]. Herein, the biologic functions of MPV17 in the development of LIHC are further studied.

In our study, systematic bioinformatics and functional experiments were conducted to assess the expression pattern, associated pathways, clinical significance, and prognostic value of MPV17 in LIHC patients. These analyses suggest that MPV17 may be related to LIHC progression and function as a novel prognostic biomarker for LIHC patients.

## 2. Materials and Methods

### 2.1. The Study on MPV17 Expression by UALCAN Database

UALCAN (The University of Alabama at Birmingham Cancer data analysis portal, https://ualcan.path.uab.edu/) [16] is a digital resource for studying cancer OMICS data. It could make a comparison with the relative gene mRNA expression levels in multiple tumors and their normal tissues. Meanwhile, the Cancer Genome Atlas (TCGA) is employed to acquire the relationship between gene mRNA levels and various clinicopathological characteristics. In this study, we analyzed the expressions of MPV17 in 33 types of tumors by UALCAN and Cox regression analysis and MPV17 expressions in LIHC groups with different clinicopathological features (sample types, tumor grade, individual cancer stages, nodal metastasis status, gender, histological subtypes, tp53 mutation status, age, and race) by TCGA.

### 2.2. The Kaplan–Meier Plotter Analysis on MPV17 and LIHC

Next, we performed a Kaplan–Meier Plotter (https://kmplot.com/analysis/) to decipher the effect of MPV17 expression on the overall survival (OS), progression-free survival (PFS), and disease-specific survival (DFS) in LIHC patients. We classified patients into high-MPV17 and low-MPV17 groups. Additionally, this classifier was verified by receiver operating curve (ROC) analysis. The area under ROC is defined as area under curve (AUC), and the higher the AUC, the greater the classifier's effect. It is defined as the area under the ROC curve, and the larger the AUC is, the better effect the classifier has. Log-rank ^*∗∗*^*P* < 0.01 and hazard ratios (HR) were calculated and displayed. Statistical difference indicated *P* < 0.05.

### 2.3. Differentially Expressed Genes (Degs) Identification and Enrichment Analyses

Through the TCGA database, we obtained 314 upregulated and 193 downregulated DEGs from high-MPV17 and low-MPV17 LIHC patients. MPV17 was in the upregulated DEGs, consistent with the findings we had before. To depict interesting gene characteristics, we conducted the widely used methods of Gene Ontology (GO) in cellular component (CC), biological process (BP), and molecular functions (MF), and Kyoto Encyclopedia of Genes and Genomes (KEGG) analyses. We used the high-throughput functional annotation bioinformatics online platform Database for Annotation Visualization and Integrated Discovery (DAVID, https://david.nicifcrf.gov/) to conduct the functional annotation and enrichment analyses (*P* < 0.05).

### 2.4. Evaluation of Risk Model

Prognosis and survival analysis can be used to study the clinical value of a gene in certain diseases. Herein, according to the median risk score, patients with LIHC were separated into two groups: high-risk and low-risk, and the corresponding survival time was displayed. Then, the OS curves of the two groups were demonstrated (median time: 2.8 and 6.6). Finally, ROC was conducted on the effect of MPV17 prediction on 1-, 3-, and 5-year prognosis.

### 2.5. Establishment of Predictive Nomogram

Firstly, univariate and multivariate analyses were conducted using the “forest plot” package to display each variable (MPV17, gender, age, pT_stage, pTNM_stage, and grade), and pertinent *P* value, HR, and 95% CI were calculated. Based on these results, nomograms were built using the “rms” package to predict 1-, 3-, and 5-year survival status. Then, the calibration curve represents the ideal prediction of the nomogram with the observation rate at the pertinent time points.

### 2.6. Correlation of MPV17 with Pathways

RNA-sequencing expression (level 3) profiles and related information for LIHC were downloaded from the TCGA dataset. The R software GSVA package was for analyzing the collected genes involved in corresponding pathways with method = “ssgsea.” Spearman's correlation was used to examine the relationship between genes and pathway scores. The abscissa depicts the gene expression distribution (its trend in the upper density curve), whereas the ordinate reflects the pathway score distribution (its trend in the right density curve). The correlation *P* value, correlation coefficient, and correlation calculation method are all shown on the top. R (4.0.3) was used to implement all of the analytic techniques and R packages. Statistical significance was defined as a *P* value of less than 0.05.

### 2.7. Immunoassay on MPV17

First, the distribution of high- and low-MPV17 immune scores in LIHC tumors and normal tissues was analyzed. For a reliable immune score assessment, a total of 6 advanced algorithms in the R package were conducted. Methodologically, the correlation between quantitative variables with abnormal distribution was described using Spearman's analysis. Finally, the relationship between MPV17 expression and the immune cell enrichment scores was demonstrated.

### 2.8. Cell Culture

LIHC cell lines, SMMC-7721 and Huh7, were acquired from the Shanghai Cell Bank, Chinese Academy of Sciences, and cultured in DMEM with 10% FBS under a 37°C incubator with 5% CO_2_.

### 2.9. Isolation and Quantification of RNA

TRIzol reagent (Invitrogen, USA) was utilized to harvest the whole RNA of the specimens or cell lines of LIHC. Harvested RNA was reverse-transcribed into cDNA utilizing the Oligo dT primer. qRT-PCR was performed utilizing the SYBR Green PCR kit (Tsingke, China) referring to the protocol on LightCycler® 480 real-time PCR (Roche, Switzerland). GAPDH was an internal reference. The relative MPV17 mRNA expression was computed by the 2^−ΔΔCT^ method.

### 2.10. Cell Transfection

We designed siRNA specific for MPV17 (si-MPV17) and then synthesized it by Ribobio (Guangzhou, China). We carried out siRNA transfection utilizing Lipofectamine RNAiMAX reagent (Thermo Fisher, MA) as instructed in the manual. Cells were collected and subjected to cell function detection 24 hours posttransfection. qRT-PCR was applied to calculate the reduction efficiency of MPV17 mRNA in SMMC-7721 and Huh7 cells.

### 2.11. Cell Proliferation Assay

The CCK-8 kit (Dojindo, China) was adopted to observe cell proliferation. In a 96-well plate, 1 × 10^3^ cells were inoculated in each well and maintained at 37°C. Cell proliferation per well was measured after 0, 24, 48, 72, and 96 h of transfection on a microtiter plate reader (Spectra Rainbow, Tecan) utilizing the Clone Select Imager System (Genetix) as the protocol depicted. All tests were repeated more than 3 times.

### 2.12. Transwell Assay

Transwell assays (24-well plate, 8 *μ*m well) were used to determine cell invasion and migration. Matrigel (BD Biosciences, CA) was precoated in the upper well of a Transwell chamber (Corning Inc., NY) for invasion detection under a 37°C incubator containing 5% CO_2_ for 1 hour. Briefly, 600 *μ*l DMEM with 10% FBS was supplemented into the lower chamber. LIHC cells in DMEM free of FBS were put into the upper chamber under a 37°C incubator for 24 hours. Then, the migrating or invading cells were rinsed with PBS, then fixed in methanol, and stained with DAPI. Cells were stochastically imaged and observed by a 100x optical microscope of individual samples.

### 2.13. Statistical Analysis

The represented data were analyzed by SPSS 20.0 (IBM, USA) plus GraphPad Prism 7 (GraphPad Software, USA). The difference in MPV17 expression in the two groups was analyzed by Student's *t*-test. The log-rank test was adopted to distinguish changes in survival time. *P* < 0.05 represented a significant difference.

## 3. Results

### 3.1. MPV17 Expressions in Pan-Cancers and the LIHC Patients with Clinicopathological Characteristics

**Figure 1:**
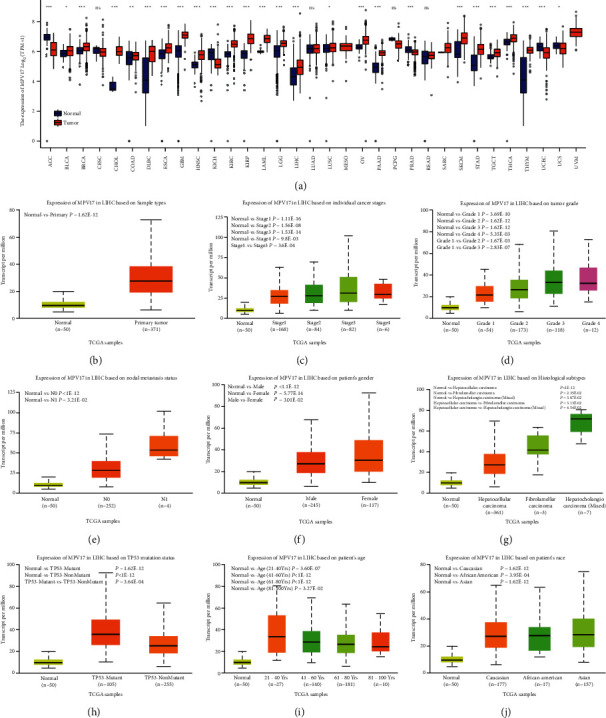
Relationship between MPV17 expression patterns and clinicopathological features in LIHC patients. (a) MPV17 expressions in pan-cancers and corresponding normal samples. (b) Comparison of MPV17 expression in primary LIHC tissues (*n* = 371) and normal tissues (*n* = 50). (c–j) MPV17 expression levels correlated with the individualized cancer stage (c), tumor grade (d), lymph node metastasis status (e), gender (f), histological subtype (g), TP53 mutation status (h), age (i), and race (j) of LIHC patients. *∗P* < 0.05; *∗∗P* < 0.01; ^*∗∗∗*^*P* < 0.001.

Through the UALCAN database, it was observed that MPV17 had a high expression in most cancers, LIHC included (Figure 1(a)), and it was highly expressed in 371 primary LIHC samples compared to 50 normal tissues (Figure 1(b)). The relationship between MPV17 expression and clinicopathological features in LIHC patients was estimated. A steady increase in MPV17 expression was observed with increased individual cancer stage (Figure 1(c)), tumor grade (Figure 1(d)), and node metastasis status (Figure 1(e)). Furthermore, higher MPV17 expression was observed in the female group than in the male group (Figure 1(f)) and hepatocholangial carcinoma in the aspect of histological subtype (Figure 1(g)). For other groups, MPV17 showed an irregular expression in the TP53 mutation group (Figure 1(h)), age (Figure 1(i)), and race (Figure 1(j)).

### 3.2. The Kaplan–Meier Plotter Analysis on MPV17 and LIHC

**Figure 2:**
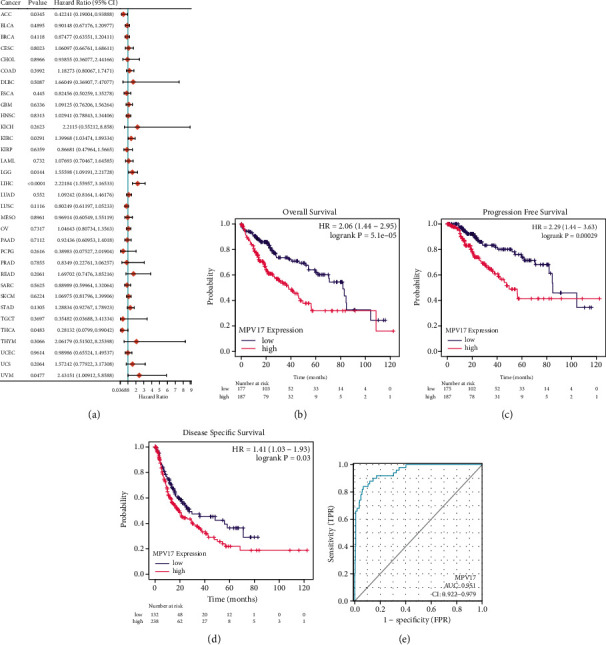
High expression of MPV17 is associated with poor prognostic status in LIHC patients. (a) Cox regression analysis on MPV17 and 33 types of tumors. (b–d) The Kaplan–Meier plotter analysis on LIHC patients with high and low MPV17 expressions in OS (b), PFS (c), and DSS (d). (e) ROC analysis on MPV17 (AUC = 0.951).

Cox regression analysis of MPV17 and various tumors showed that LIHC was statistically significant with MPV17 (*P* < 0.0001, Figure 2(a)). Then, we performed a Kaplan–Meier survival analysis on MPV17 in LIHC. Given the median expression of MPV17 mRNA, we classified the patients into two groups based on MPV17 expression: high and low, respectively, which was verified by ROC analysis on MPV17 (Figure 2(e), AUC = 0.951). As shown in Figure 2(b), patients with higher MPV17 expression exhibited shorter OS times relative to patients with low MPV17 expression. In addition, increased MPV17 expression also demonstrated significantly reduced PFS (Figure 2(c)) and DSS time (Figure 2(d)), confirming that increased MPV17 expression was a risk factor for the patient's prognostic status.

### 3.3. GO and KEGG Analyses on the Identified DEGs

**Figure 3:**
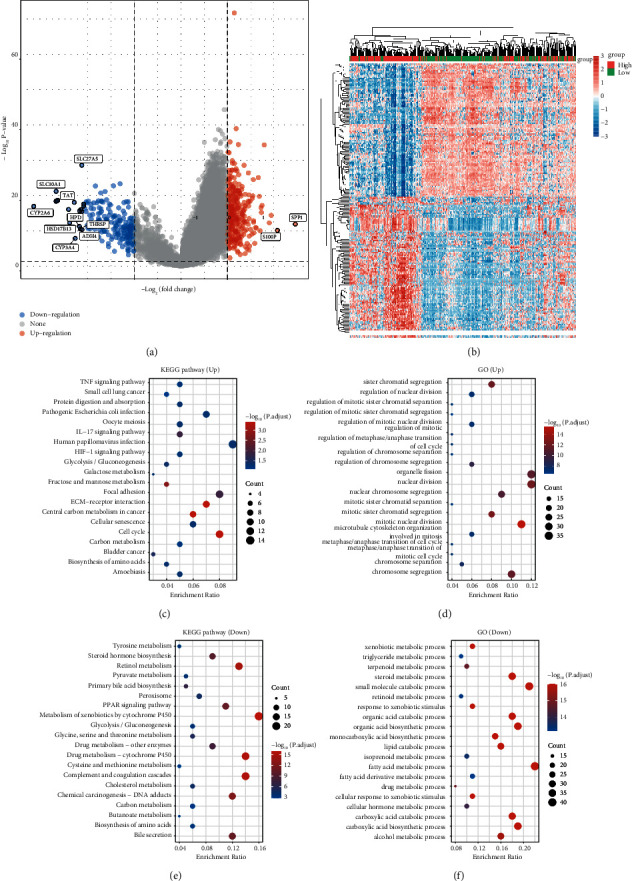
Identification of DEGs and GO, and KEGG pathway enrichment analysis. (a) Volcano plot of DEGs. (b) Heatmap of DEGs. (c, d) KEGG and GO enrichment analysis on upregulated DEGs. (e, f) KEGG and GO enrichment analyses on downregulated DEGs.

We acquired 314 upregulated and 193 downregulated LIHC-related DEGs based on MPV17 expression from the TCGA database. The volcano plots and heat maps of DEGs were mapped (Figures 3(a) and 3(b)). Then, GO and KEGG analyses were conducted on them, respectively. The upregulated DEGs were in KEGG enriched in TNF signaling pathways, small-cell lung cancer, etc. (Figure 3(c)), and in sister chromatid segregation and regulation of nuclear division in GO (Figure 3(d)). Downregulated DEGs were enriched in KEGG in tyrosine metabolism, steroid hormone biosynthesis, etc. (Figure 3(e)), and xenobiotic metabolic process and triglyceride metabolic process in GO (Figure 3(f)).

### 3.4. MPV17 Had Prognostic Value for LIHC Patients

**Figure 4:**
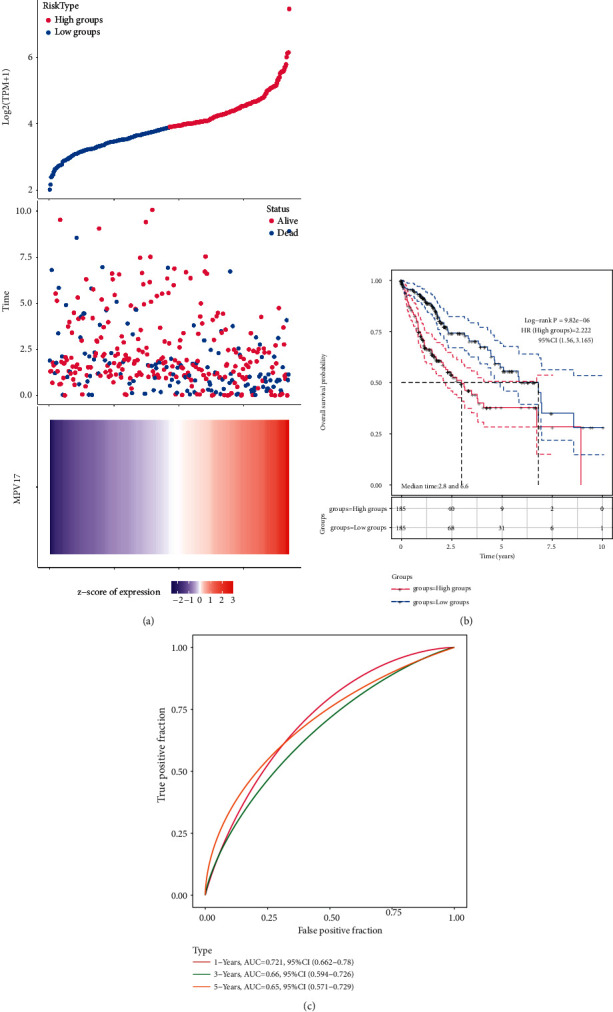
Risk model for MPV17 and LIHC. (a) The relationship between MPV17 expression, survival time, and survival status. (b) Overall survival probability of MPV17 expression in high-risk and low-risk groups. (c) ROC analysis on MPV17 and LIHC prognosis at 1, 3, and 5 years.

We assigned each patient a risk score and separated them into high-risk (*n* = 185) and low-risk (*n* = 185) groups.  Figure 4(a) shows the survival time of patients, and the expression of MPV17 had a positive relationship with a risk score. In Figure 4(b), when compared to the low-risk group, the high-risk group had a poorer overall survival rate. As the ROC analysis shown, MPV17 had the best predictive potential in 1-year overall survival (Figure 4(c), AUC = 0.721).

**Figure 5:**
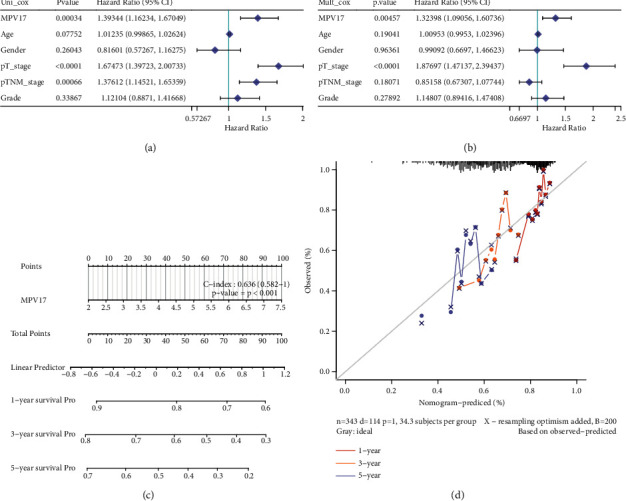
Predictive nomogram analysis of MPV17. (a, b) Cox analysis on MPV17 expression and clinical characteristics in LIHC. (c) Nomogram predicts 1-, 3-, and 5-year overall survival of HCC patients. (d) Calibration curve represents the ideal nomogram.

The univariate and multivariate analyses were initially established to ensure the precision of prediction. MPV17 and pT_stage had significant relation with LIHC prognosis (Figures 5(a) and 5(b)). Then, a nomogram on MPV17 to predict 1-, 3-, and 5-year prognosis in LIHC patients was mapped (Figure 5(c)). The calibration plot for prognostic prediction showed the results of the MPV17 nomogram were in consistence with the actual results (Figure 5(d)). These results indicate that MPV17 could serve as an independent prognostic biomarker.

### 3.5. Pathway Correlation Analysis on MPV17

**Figure 6:**
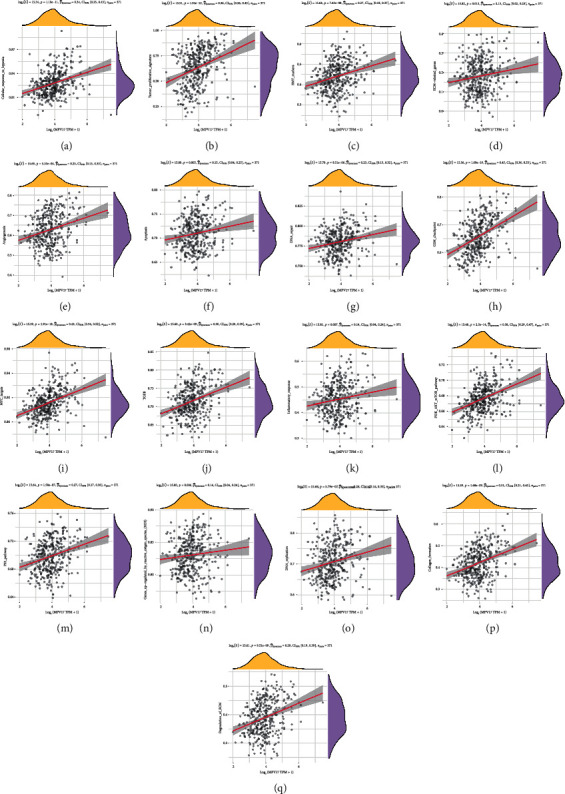
Pathway correlation analysis on MPV17. (a–q) Spearman's correlation analysis between MPV17 and 17 pathways' scores.

In the gene-pathway correlation analysis, 19 pathways related to LIHC were chosen, while we found that MPV17 was positively correlated with 17 pathways, including Cellular_response_to_hypoxia, Tumor_proliferation_signature, EMT_markers, ECM-related_genes, Angiogenesis, Apoptosis, DNA_repair, G2M_checkpoint, Inflammatory_response, PI3K_AKT_mTOR_pathway, P53_pathway, MYC_targets, TGFB, Genes_upregulated_by_reactive_oxigen_species_(ROS), DNA_replication, Collagen_formation, and Degradation_of_ECM. MPV17 had the strongest correlation with the G2M_checkpoint (Figures 6(a)–6(q)).

### 3.6. Correlation Analysis of MPV17 and Immune Cells

**Figure 7:**
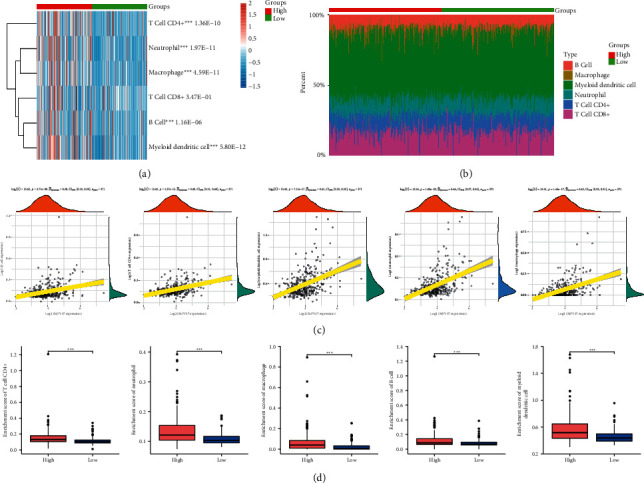
Correlation analysis between MPV17 and immune cells. (a) Different expressions of immune cells in high-expressed and low-expressed MPV17 groups. (b) The percentage abundance of tumor-infiltrating immune cells in each sample. (c) Spearman's correlation analysis between MPV17 and immune cell expression. (d) Enrichment scores of immune cells in high- and low-expressed MPV17 groups. Immune cells with high MPV17 expression also had higher enrichment scores.  ^*∗∗∗*^*P* < 0.001.

Different expressions of immune cells in high-expressed and low-expressed MPV17 groups are shown in Figure 7(a), while the results of T cell CD8+ did not have statistical significance.  Figure 7(b) demonstrates the percent abundance of LIHC immune cells in high-expressed and low-expressed MPV17. MPV17 had a positive correlation with B cells, T cell CD4+, myeloid dendritic cells, neutrophils, and macrophages. Among them, MPV17 had the strongest correlation with neutrophil (Figure 7(c)). Next, the enrichment scores of immune cells with high MPV17 expression were higher than those of immune cells with low MPV17 expression (Figure 7(d)).

### 3.7. Silenced MPV17 Weakened the Cell Proliferation, Migration, and Invasion Abilities

**Figure 8:**
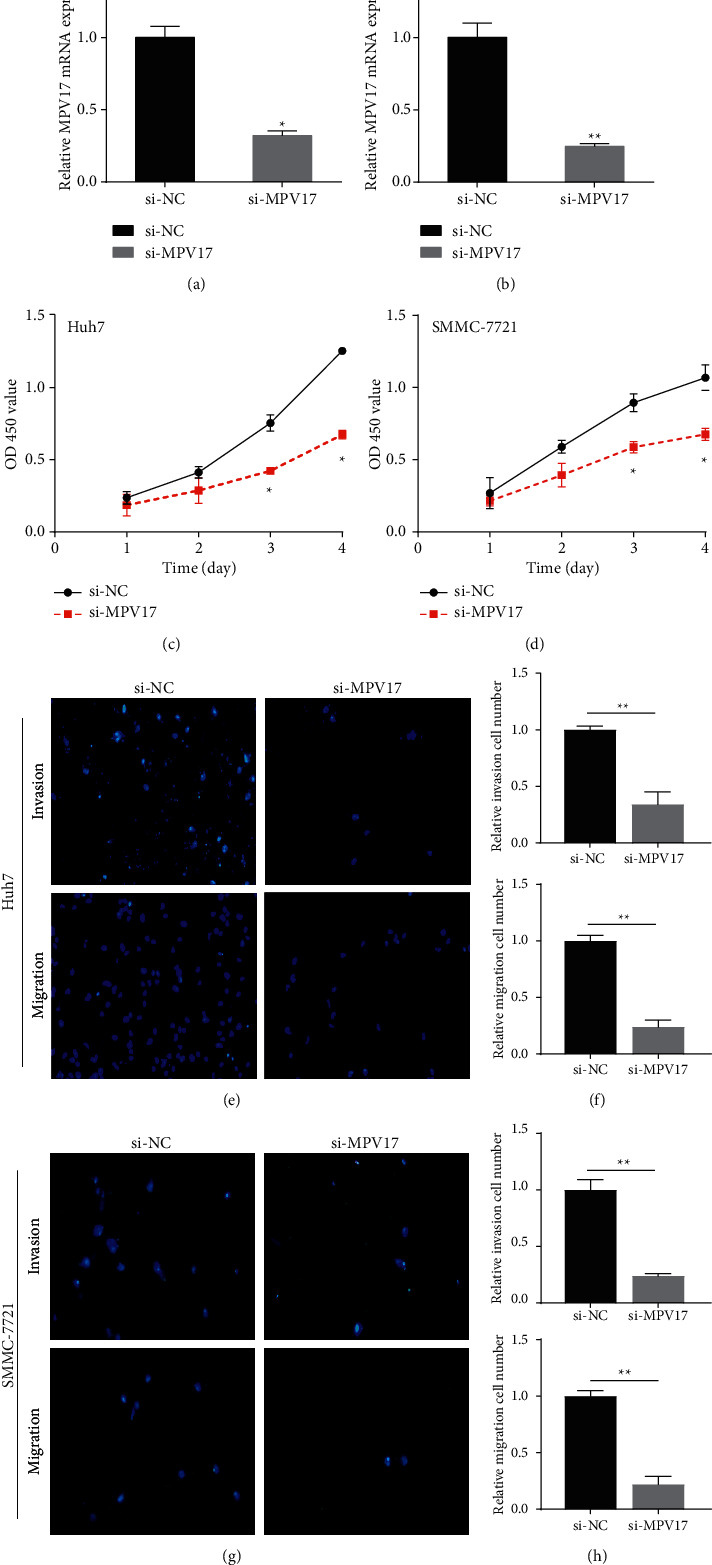
MPV17 knockdown weakened cell proliferation, migration, and invasion abilities. (a, b) Transfection of si-MPV17 in SMMC-7721 and Huh7 cells. (c, d), Knockdown of MPV17 led to the suppression of cell proliferation ability. (e, h), MPV17 knockdown resulted in reduced cell migration and invasion abilities. ^∗^*P*<0.05; ^∗∗^*P*<0.01.

In functional assays, we validated the effects of MPV17 knockdown on cell proliferation, migration, and invasion. MPV17 siRNAs were transfected into SMMC-7721 and Huh7 cells. Subsequently, we conducted qRT-PCR to observe the reduction efficiency of MPV17 (Figures 8(a) and 8(b)). CCK-8 results indicated that downregulating MPV17 greatly impeded SMMC-7721 and Huh7 cell proliferation (Figures 8(c) and 8(d)). Transwell assays displayed that knockdown of MPV17 expression dramatically repressed cell migration and invasion abilities (Figures 8(e)–8(h)). Our data indicated that MPV17 displayed as a promoter in the cell proliferation, migration, and invasion of LIHC.

## 4. Discussion

Currently, it is not clear about the pathogen and the detailed mechanism of primary liver carcinoma. People generally think its onset is a complicated process with multiple factors and steps influenced by the environment and diet [17]. Epidemiological research data and some studies reveal many contributors induce liver carcinoma, including hepatitis B and hepatitis C virus infection, aflatoxin, ethanol, liver cirrhosis, nitrosamines, and so on [18–21]. All of them exhibit a relationship with the onset of liver carcinoma. Considering the different stages of liver carcinoma, the personalized integrated strategy is pivotal to ameliorating the curative efficacy. Treatment including surgery, radiofrequency, laser, chemotherapy, and others are usually unsatisfactory [22–24]. New biomarkers and targets still need to be found. In our paper, we investigated the expression and clinical significance of MPV17 in LIHC through bioinformatics analysis and functional experiments.

MPV17, also named SYM1, CMT2EE, and MTDPS6 is an inner membrane protein of mitochondrial participating in ROS metabolism [25, 26]. At present, there are few studies about it. Many researchers discuss its relationship with mitochondrial DNA depletion syndrome (MDDS). For example, Löllgen and Weigher reported that mutations of MPV17 could cause MDDS [27]. Kim et al. analyzed the relationship of mutated MPV17 with hepatocerebral MDDS patients [28]. Besides, Canonne et al. held a controversial view on the effect of MPV17 on carcinoma cell proliferation [29]. It still needs more research to evaluate the clinical significance and the possible mechanism of MPV17 in the development of LIHC. Through UALCAN and TCGA databases, we found that MPV17 was upregulated in most tumors, and its expression in LIHC patients had a great association with carcinoma stages, tumor grades, nodal metastasis status, gender, histological subtypes, and TP53 mutation status, implying that MPV17 might act as an oncogene in LIHC. This conclusion was verified by our functional experiments. Cell function experiments demonstrated silenced MPV17 in LIHC cells inhibited cell proliferation, migration, and invasion. These findings indicate that MPV17 might play an oncogenic role in LIHC. In addition, MPV17 expression and LIHC prognosis were studied by the Kaplan–Meier curve. It was demonstrated that elevated MPV17 expression endangered the prognosis of LIHC patients.

In addition, we divided LIHC samples based on MPV17 expression in the TCGA database and finally got 314 upregulated and 193 downregulated DEGs. MPV17 was in the upregulated group. We also conducted a functional enrichment analysis to better explore the interconnections among DEGs. GO analysis showed DEGs were related to regulation of mitotic sister chromatid separation, sister chromatid segregation, organic acid catabolic process, and drug metabolic process. As in previous studies, Vander Heiden and DeBerardinis showed that transformed cells change their metabolism to facilitate tumorigenesis. Specific metabolic activities can play a direct role in tumor transformation or promote biological mechanisms in tumors [30]. In addition, McGranahan et al. show that cancer chromosomal instability leads to an increased rate of change in chromosome number and structure and creates intratumoral heterogeneity [31]. This may suggest that many of the DEGs in this study are involved in the metabolism of functional molecules in vivo and may be involved in the chromosome replication pathway during cell proliferation. KEGG analysis revealed that DEGs were related to several cancer-related pathways, such as small cell lung cancer, bladder cancer, tyrosine metabolism, protein digestion, absorption, etc. Digestion and absorption of protein have been verified to participate in the development of cancer. Besides, we also performed pathway correlation analysis on MPV17, and it was closely related to the G2M_checkpoint pathway involved in cancer progression. Oshi et al. demonstrated the prognostic value of the G2M cell cycle pathway score in estrogen receptor-positive breast cancer metastasis [32]. Liu et al. found that aloperine induced apoptosis and G2/M cell cycle arrest in hepatocellular cancer cells through inhibition of the PI3K/Akt signaling pathway [33]. In a word, we suppose that MPV17 could affect the initiation and progression of LIHC through the above biological terms and pathways, particularly the G2M_checkpoint pathway.

Cell immunotherapy is to collect the body's own immune cells (like peripheral blood mononuclear cells), make them multiply by thousands of times after *in vitro* culture, and then back to the human body to kill pathogens in blood and tissue cells, cancer cells, and mutations. Thus, the body's immune ability is activated and strengthened to achieve the prevention of tumor recurrence and metastasis [34]. Herein, immunoassay analysis exhibited that MPV17 had a positive correlation with B cells, T cell CD4+, myeloid dendritic cells, neutrophils, and macrophages. The expression of immune cells would elevate with the increase of MPV17 expression, and the enrichment scores of immune cells with high MPV17 expression were higher than those of immune cells with low MPV17 expression. These results may imply that MPV17 could be one new immunotherapy target in LIHC treatment.

## 5. Conclusion

In summary, we first find and verify that MPV17 is an oncogenic gene in LIHC and it has potential clinical value in its prognosis through multiple bioinformatics analyses and functional experiments. Our results indicate MPV17 could be a prospective biomarker for LIHC prognosis and a candidate target for LIHC immunotherapy.

## Data Availability

The datasets used and/or analyzed during the current study are available from the corresponding author on reasonable request.
